# Comparing Diagnosis and Treatment of Pulmonary Hypertension Patients at a Pulmonary Hypertension Center versus Community Centers

**DOI:** 10.3390/diseases10010005

**Published:** 2022-01-07

**Authors:** Hollie Saunders, Scott A. Helgeson, Ahmed Abdelrahim, Kathleen Rottman-Pietrzak, Victoria Reams, Tonya K. Zeiger, John E. Moss, Charles D. Burger

**Affiliations:** 1 Department of Pulmonary Medicine Mayo Clinic, 4500 San Pablo Road, Jacksonville, FL 32224, USA; helgeson.scott@mayo.edu (S.A.H.); ahmedrahim02@gmail.com (A.A.); zeiger.tonya@mayo.edu (T.K.Z.); moss.john@mayo.edu (J.E.M.); burger.charles@mayo.edu (C.D.B.); 2 Department of Pharmacy Mayo Clinic, 4500 San Pablo Road, Jacksonville, FL 32224, USA or rottmanpietrzak.kathleen@mayo.edu (K.R.-P.); victoria.r3@hotmail.com (V.R.)

**Keywords:** pulmonary hypertension, pulmonary hypertension center, medication management

## Abstract

Once patients are diagnosed with pulmonary hypertension it is important to identify the correct diagnostic group as it will have implications on the disease state management. Pulmonary hypertension is increasingly diagnosed and treated in general medical practices; however, evidence-based guidelines recommend evaluation and treatment in pulmonary hypertension centers for accurate diagnosis and appropriate treatment recommendations. We conducted a retrospective cohort study of 509 random patients 18 years and older who were evaluated in our pulmonary hypertension clinic from January 2005 to December 2018. 68.4% (*n* = 348) had their diagnostic group clarified or changed. Pulmonary hypertension was deemed an incorrect diagnosis in 12.4% (*n* = 63). A total of 114 patients (22.4%) had been initiated on pulmonary hypertension specific treatment prior to presentation. Pulmonary hypertension specific medication was stopped in 57 (50.0%) cases. The estimated monthly saving of the stopped medication based on wholesale acquisition costs was USD 396,988.05–419,641.05, a monthly saving of USD 6964.70–7362.12 per patient. Evaluation outside of a pulmonary hypertension center may lead to misdiagnosis and inappropriate or inadequate treatment. Pulmonary arterial hypertension directed therapy improves median survival, but inappropriate therapy may cause harm; therefore, patients benefit from a specialized center with multiple resources to secure an accurate diagnosis and tailored treatment for their condition.

## 1. Introduction

Evidence-based guidelines for the evaluation and treatment of pulmonary hypertension (PH) have evolved considerably over the last decade. The most recent guideline from the World Symposium on PH (WSPH) in 2018 recommended redefining PH from an elevated mean pulmonary artery pressure (mPAP) of 25 mmHg to a lower threshold of >20 mmHg [1]. The intent was earlier detection in the disease course; however, the frequency of diagnosis may increase, perhaps with less specificity. Notably, the diagnosis of PH encompasses a wide range of pathophysiology separated into five broad diagnostic groups, each with its own treatment pathway [2,3]. Diagnostic PH groups are based on the WSPH classification with group 1 consisting of pulmonary arterial hypertension (PAH), group 2 PH secondary to left heart disease, group 3 PH caused by lung diseases or hypoxia, group 4 due to chronic thromboembolic pulmonary hypertension (CTEPH), and group 5 containing PH due to uncertain causes [1]. 

Determination of the correct diagnostic group has direct implications on prognosis and management and is particularly important for group 1 PAH where a median survival without treatment is 2.8 years [4]. Currently, PH specific therapy is only indicated in group 1 PAH, select cases of group 3 PH due to interstitial lung disease (ILD) as of 1 April 2021, and in group 4 CTEPH [5,6]. Goal-directed therapy improves 1-year and 5-year survival in group 1 to approximately 86% and 61%, respectively [7]. Evidence-based guidelines encourage early referral to a PH specialty center to ensure optimum outcomes [5,6]. As such, the Pulmonary Hypertension Association established designated PH Comprehensive Care centers (PHCC) to provide specialized evaluation and treatment [8]. Fortunately, increased awareness and medical treatment options, as well as availability of transthoracic echocardiography and bedside ultrasound, may promote more frequent identification of PH in the general medical practice; however, treatment should only be initiated after confirmatory right heart catheterization (RHC) [5]. The balance of distribution of PH care between expert centers and general medical practices may result in varied adherence to guidelines [8]. A PAH quality enhancement initiative identified that guideline recommended tests were only being performed six percent of the time in a community practice [9]. In addition, PH centers have seen a trend of referrals of patients already on specific therapies [10] and, at times, inappropriately. Specialized centers not only maintain the lowest complication rates with the best outcomes, but also greater patient satisfaction and value for health care payers [8]. 

As an accredited PHCC, we aimed to show an improved diagnostic and appropriate treatment rate following evidence-based guidelines for diagnosis and treatment. Additionally, we wanted to show a positive patient impact on patient medication costs. 

## 2. Methods

### 2.1. Study Cohort Selection

A retrospective cohort study was conducted on a randomly selected group of patients newly referred to a certified PHCC from January 2005 to December 2018. Over this time, each patient seen in our PH clinic was added daily to our quality database. Patients were randomly selected after being manually placed in alphabetical order of their last name, starting at the letter “A”, and reviewing consecutive patients. Selection was continued to obtain at least 500 patients. All patients 18 years and older were included, but they were excluded if they did not complete the recommended testing and/or follow-up appointments. The Mayo Clinic Institutional Review Board approved the study (IRB 18-010856).

### 2.2. Defining PH Testing and Groups

As a PHCC, the most up to date diagnostic and management guidelines were followed. At the initial visit, all medical records were reviewed by the PH specialists (CDB, JEM) and additional testing or repeat testing was performed if the quality of the test was deemed to be inadequate. 

The evaluation of newly referred patients consisted of following diagnostics: laboratory work (complete blood count, basic metabolic profile, liver function tests, thyroid function tests [11], antinuclear antibody test [12], anti-CCP antibody, human immunodeficiency virus test in patients at risk by history [13], NT-pro brain natriuretic peptide (BNP) or BNP, arterial blood gas, pulmonary function test (body plethysmography, spirometry, and diffusion capacity), six-minute walk test, cardiopulmonary exercise testing most often with SHAPE-HF™ (Shape Medical Systems, Inc., Saint Paul, MN, USA), overnight oximetry, and if clinically indicated overnight polysomnography, electrocardiogram, echocardiogram with agitated saline, chest radiograph, high-resolution computed tomography of the chest, ventilation perfusion (VQ) scan (computed tomography pulmonary angiography if VQ positive), RHC with acute vasoreactivity testing, and left heart catherization if indicated [5,14,15]. The diagnosis of PH was established based on current guidelines [1,5]. To account for the 2018 WSPH change in hemodynamic definition, those patients seen prior to the guideline change were deemed to have PH if they had a mPAP of >25 mmHg and those seen after the guideline change were deemed to have PH if they had a mPAP of >20 mmHg. 

Data for this study were collected by review of the patient’s electronic medical record for the patient demographics, co-morbid medical conditions, date of initial visit, diagnostic group of PH at initial visit, functional class, PH specific medications, diuretics or calcium channel blockers, test results as described above, subsequent follow-up date, whether there was a change or confirmation in the diagnostic group, and whether there was change (addition or removal) in PH medications. If patients were deemed not to have PH then an alternate diagnosis was collected.

The diagnostic groups of PH at the initial visit were defined as the five groups of PH with a separate group, designated “undifferentiated PH”, as many patients were referred without a specific diagnostic group identified. A change in diagnosis was defined as changing the referral diagnostic group at the initial visit, to a different group at the subsequent follow-up visit. A change in diagnosis also included clarifying from the undifferentiated PH designation to a specific diagnostic group, or if PH was excluded. The method by which PH was excluded was either a clinical evaluation including RHC or clinical evaluation without RHC. Circumstances for the latter were based on clinical judgment by the PH specialist that generally included a negative history for PH risk factors including connective tissue disease, venous thromboembolism, hematologic disorder, HIV, anorexins or illicit drug use, sleep disordered breathing, thyroid disease, and liver or lung disease. A credible alternative explanation for the symptoms was required. Patients in whom PH was excluded without RHC had normal echocardiographic findings defined as the absence of any of the following: increased right heart size, elevated right heart pressures including mean pulmonary pressure [16], reduced right ventricular function, or significant tricuspid regurgitation [5]. In most cases, patients had further evaluation with a submaximal heart and pulmonary stress by SHAPE-HF™, pulmonary function testing, and a six-minute walk. Findings that indicated a low likelihood of PH on these tests were a normal diffusion capacity (DLCO), good performance on six-minute walk without evidence of desaturation, and a breathing efficiency (minute ventilation/carbon dioxide production, VE/VCO_2_) < 30 with an end exercise end-tidal CO_2_ (PETCO_2_) of >37 mmHg on submaximal exercise testing. After the initial history and physical, the PH specialist reviewed the screening test results and determined that the patients were unlikely to have PH without the need for invasive RHC. Examples of referral reasons for patients deemed not to have PH without invasive testing included incidentally noted enlarged pulmonary artery on imaging, self-referral, and prior pulmonary embolus.

### 2.3. PH Medications and Costs

Specific PH medications recorded for the analysis included endothelin receptor antagonists (ambrisentan, bosentan, and macitentan), phosphodiesterase type 5 inhibitors (sildenafil and tadalafil), guanylate cyclase stimulator (riociguat), prostacyclin receptor agonist (selexipag), and prostacyclin analogues (epoprostenol, treprostinil, and iloprost). A calcium channel blocker was also considered PH specific therapy if there was a documented positive vasoreactivity test. The cost of each PH drug was obtained using the average selling price sourced from Lexicomp. For our calculations the cost of a 30-day supply was used, based on average selling price and average daily dose, to highlight an average monthly cost. Using the information in Supplemental Table S1 we calculated the combined cost of both the stopped and initiated medications (listed in results). For medications that have several different prices listed for a 30-day supply, such as epoprostenol and selexipag, and average of the cost was used. The calculated cost is displayed as a range that accounts for the differences in cost between brand and generic. The lower end of the range reflects the calculated generic cost and the higher end of the range reflects the brand cost. The calculated costs are reflective of medication cost within the United States and may be under or overestimated compared to the cost of the same medication in different countries. 

### 2.4. Statistical Analysis

A descriptive analysis was performed using SPSS^®^ V25 (IBM^®^; Armonk, NY, USA). Categorical data were displayed as the number (percentage of total) and continuous data were displayed as median (interquartile range). The median with interquartile range was used, because after a visual inspection of the data, it was not found to be normal. 

## 3. Results

A total of 509 patients were analyzed who were mostly white and female with an age range over two decades as in Table 1. Most were functional class III and co-morbidities were common.

**Figure 1:**
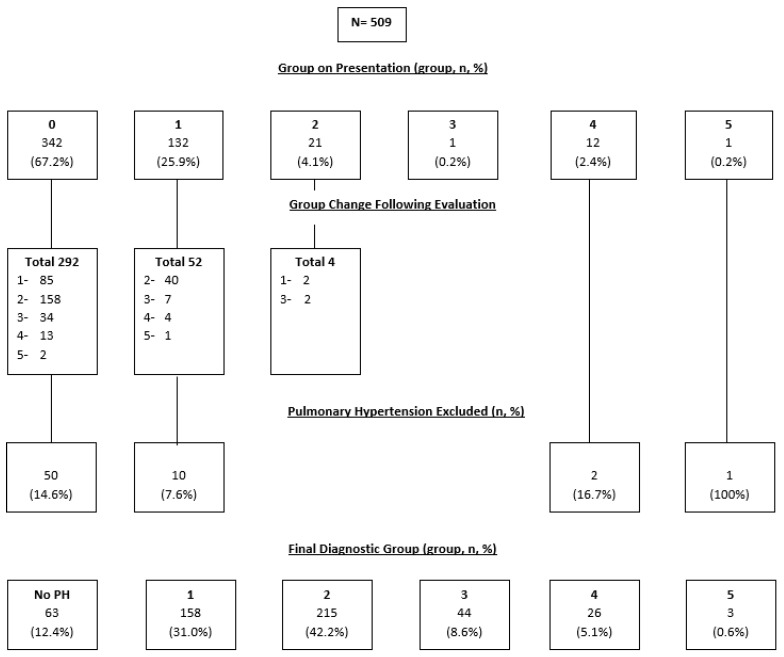
Diagnostic accuracy of 509 randomly selected patients beginning with the referral diagnosis, noting that 342 patients were not classified in a pulmonary hypertension diagnostic group, referred to as group “0” in the figure. The “Group Change Following Evaluation” displays a subset of referral diagnosis and the reclassification into group 1, 2, 3, 4, or 5 as determined by the guideline-based evaluation in the pulmonary hypertension clinic.

The initial, revised, and final diagnosis are displayed in Figure 1. At the initial visit, more than two-thirds of the entire cohort were characterized by the referring provider as undifferentiated PH and approximately one-quarter initially characterized as group 1 PAH. Groups 2, 3, 4, and 5 comprised the remaining 7%. After evaluation, most of the patients were reclassified as follows: group 1, approximately 30%; group 2, about 40%; group 3, just under 10%; and group 4, at 5% (see “Final Group” in figure). Overall, PH was excluded in over 10% of the total cohort. Of the 63 excluded patients, 17 (27.0%) patients were excluded based on normal pulmonary pressures measured on RHC, and 46 (73.0%) were excluded based on clinical evaluation without RHC. For the patients who were excluded from having PH, the most common alternative diagnosis was restrictive ventilatory disease (including ILD, obesity, chest wall deformity, and neuromuscular weakness) in 13 (20.6%) patients, followed by obstructive lung disease (including chronic obstructive pulmonary disease and asthma) in 12 (19.0%) patients, and heart failure with preserved ejection fraction in 7 (11.1%) patients. Seven patients were those with a history of pulmonary embolus referred to rule out CTEPH. Other alternative diagnoses included high cardiac output states as seen with end stage liver disease, untreated OSA, and congenital heart conditions such as an atrial septal defect. Most patients (348 of 509, 68.4%) had their PH diagnostic group changed from their initial group on presentation. 

Of the initially suspected group 1 cohort, only 70 of 132 (53.0%) were confirmed to have true group 1 PAH. An additional 85 patients from the undifferentiated PH group and 2 patients from the suspected group 2 PH group were reclassified and diagnosed as group 1 PAH, to comprise the final group of 158 of 509 (30.6%) patients with confirmed group 1 PAH. Several of the initially categorized group 1 patients (53 of 132, 40.2%) were reclassified, most to group 2 (40 of 53, 75.5%). In addition, some were determined not to have PH (10 of 132, 7.6%).

Breakdown of the undifferentiated group at initial presentation, revealed that 292 of 342 (85.4%) were reclassified into a new group: group 1 (85 of 292, 29.1%), group 2 (158 of 292, 54.1%), group 3 (34 of 292, 11.6%), group 4 (13 of 292, 4.5%), and group 5 (2 of 292, 0.7%). Notably, 50 of 342 (14.6%) undifferentiated patients were determined to not have PH. The final redistribution of the diagnostic groups after completing evaluation is displayed in Figure 1. 

Importantly, all confirmed, and newly diagnosed group 4 patients were discussed at a multidisciplinary CTEPH conference for consideration of pulmonary thromboendarterectomy. Typically, the case is presented by one of our PH specialists to a team that includes an interventional cardiologist and radiologist, a thoracic surgeon experienced in pulmonary thromboendarterectomy, diagnostic radiology and lung transplant. Of the 26 group 4 CTEPH patients, 11 (42.3%) were deemed inoperable either due to clot characteristics (distal clot or small clot burden) or co-morbidities such as severe ILD, active solid organ cancer, or dementia. A total of seven (26.9%) patients underwent pulmonary thromboendarterectomy. Eight patients either declined the procedure or further evaluation. 

A total of 114 (24.4%) patients were already prescribed PH specific medication on presentation. Breakdown by diagnostic group revealed 104 patients were previously diagnosed with suspected group 1 PAH, 5 patients with suspected group 2, and 5 patients with suspected group 4. Of the 114 patients already on PH specific medication, 27 (5.3%) had an additional PH medication added to achieve combination therapy, 2 of which had more than one PAH medication added. Ninety-five patients (18.6%) were initiated on PH specific medication at follow up, eight (1.8%) of these patients were started on combination therapy at initiation. All patients who were started on PH medication were diagnosed with either group 1 PAH or group 4, with the exception with one patient with predominantly group 3 disease with a noted high pulmonary vascular resistance on RHC. The patient was prescribed inhaled treprostinil. A total of 131 medications were initiated in 121 patients and included sildenafil (*n* = 49, 37.4%), ambrisentan (*n* = 18, 13.7%), inhaled treprostinil (*n* = 17, 13.0%), tadalafil (*n* = 17, 13.0%), epoprostenol (*n* = 12, 9.2%), riociguat (*n* = 6, 4.5%), subcutaneous treprostinil (*n* = 4, 3.1%), macitentan (*n* = 4, 3.1%), bosentan (*n* = 2, 1.5%), intravenous treprostinil (*n* = 1, 0.8%), and selexipag (*n* = 1, 0.8%). The total 30-day supply cost of the started medication was calculated as USD 1,105,455.45–1,186,397.25. The method by which the cost was calculated is described in the methods section. When divided by patient (*n* = 121) and converted to per day this amounts to a daily cost of USD 304.53–326.83 per patient. 

Specific PH medications were stopped in 57 (11.2%) patients. A total of 60 medications were discontinued, noting that some patients were on combination therapy. The discontinued medications included sildenafil (*n* = 34, 56.7%), tadalafil (*n* = 8, 13.3%), and riociguat (*n* = 7, 11.7%), ambrisentan (*n* = 6, 10.0%), inhaled treprostinil (*n* = 2, 3.3%), macitentan (*n* = 2, 3.3%), and selexipag (*n* = 1, 1.7%). The sum of the monthly medication cost for all the discontinued medications, listed above, came to USD 396,988.05–419,641.05. We divided this total by the number of patients with medication stopped (*n* = 57), to obtain a monthly average saving of USD 6964.70–7362.12 per patient who had a medication stopped. If divided over 30 days, this amounts to a USD 232.16–245.40 daily saving per patient. The average selling price for PH-specific medications is shown in Supplemental Table S1. 

## 4. Discussion

Two factors have led to increased detection of increased pulmonary pressures: increased awareness of the disease and increased utility of echosonography. More widespread testing may increase the number of patients diagnosed with PH; however, additional evaluation is required to avoid over or misdiagnosis. A misdiagnosis of PH due to a failure to complete a comprehensive work up has been described in the literature [9,17] and results in patient receiving inappropriate and expensive therapy. We found that 68.4% of patients referred to us were diagnosed with a different than suspected PH group and 12.4% with no PH. Our results showed a larger cohort of misdiagnosed patients compared to the multi-center study conducted by Deano et al. in 2013 [17], as that study identified misdiagnosis in 33% of patients. The difference may be due to our larger patient population (509 versus 140). It may also reflect increased screening for and detection of PH. The latter is consistent with larger proportion of referred patients with undifferentiated PH at 67.2% in the current study, compared to Deano et al. at 30%. Of note, the patients referred to our center with undifferentiated PH were generally because a complete assessment had not been performed. 

Interestingly, the next largest referral group were those with suspected group 1 PAH. More than three-quarters were already on PH specific medication on initial evaluation; however, after full evaluation, only 53.0% of them were deemed to have group 1 PAH. In addition, 10 patients without suspected PAH presented on PH specific medication. In total, 57 patients were on inappropriate and expensive PAH therapy. The list of discontinued medications is detailed in the results and amounted to monthly average saving of USD 6964.70–7362.12 per patient, USD 232.16–245.40 per day per patient. When considering specific medications, we noted that the most frequently discontinued medication was sildenafil followed by tadalafil. As can be seen from Supplemental Table S1, these are some of the least expensive PH medications. They are also oral, easily obtainable, and with relatively few side effects. Given these factors physicians may consider starting these medications in PH groups other than group 1 PAH to see if some benefit can be achieved. Especially, in the cases of group 2 PH, if other therapeutic options are exhausted. This practice is one that has not been shown to improve clinical status or exercise capacity, and may actually cause harm [18]. 

Despite insurance and financial assistance programs, PH patients still report a significant financial burden associated with their disease [19]. The identification of inappropriate treatment and its associated cost can have huge changes in a patient’s quality of life, where financial strain plays a large role. Even in the cases of commonly used medications financial burden leads to emotional strain as well as poor compliance. Empiric treatment is not advisable avoid unnecessary adverse effects and expense. It is extremely important that prescribing be based on a thorough and complete assessment, preferably at a PHCC [5]. 

Treatment based on published guidelines for those patients in whom group 1 PAH was confirmed should also be highlighted. In total, 122 patients had PAH medication initiated or added. Patients on monotherapy may need escalation to combination therapy, as evidence indicates that early introduction improves clinical outcomes and reduces hospitalizations due to worsening PAH [6,20,21,22,23]. Often the cost of the PAH medication is offset by the reduced need for inpatient hospitalization, thereby balancing the total healthcare costs [22,23]. For example, Burger et al. demonstrated that most patients were initiated on monotherapy, but that early initiation of combination therapy could reduce PAH-related hospitalizations [23], further reducing overall healthcare costs. 

Finally, it is important to highlight the potential benefit offered by a multidisciplinary approach available in a PHCC. For example, group 4 CTEPH patients are reviewed for potential interventional treatment for their disease. Pulmonary thromboendarterectomy was performed successfully in seven patients, most of whom no longer required ongoing PH therapy. Balloon pulmonary angioplasty was not available in our center during the timeframe of this study, but now patients who are not surgical candidates due to the presence of distal clot or small clot burden are offered both medical therapy and pulmonary balloon angioplasty as indicated by current guidelines [24].

Our study is not without limitations. The study was conducted in a single center and may have suffered from the data collection limitations commonly associated with retrospective studies. It is possible that some of our findings may be under-estimated, especially in the case of medication changes. We function as a referral center and often our recommendations will be reported to the referring physician. The changes are then made outside of the institution where the patient is followed. 

In conclusion, our study has highlighted that evaluation and management outside of a PH center can lead to misdiagnosis and inappropriate treatment of PH. Correctly classifying the appropriate diagnostic group is critical to determining best treatment. These complex patients benefit from evaluation in a specialized center with expertise and committed resources to establish an accurate diagnosis and effective treatment for their condition. The specialty center evaluation may also result in more appropriate use of healthcare resources required for PH treatment.

## Figures and Tables

**Table 1:** Patient Demographics.

		Total (*n* = 509)
Age	*n*	509
	Median	63.6
	Percentiles (25th, 75th)	55, 74
Sex	Male	173 (34.0%)
	Female	336 (66.0%)
Race	White	410 (80.6%)
	Black	87 (17.1%)
	Asian	7 (1.4%)
	American Indian or Alaskan Native	2 (0.4%)
	Unknown	3 (0.6%)
Ethnicity	Non-Hispanic	491 (96.5%)
	Hispanic	18 (3.5%)
Function Class	1	13 (2.6%)
	2	70 (13.8%)
	3	283 (55.6%)
	4	18 (3.5%)
Co-morbidities at initial visit	COPD	103 (20.2%)
	Interstitial Lung Disease	57 (11.2%)
	Sleep Apnea	184 (36.1%)
	History of PE or DVT	78 (15.3%)
	Hypertension	274 (53.8%)
	Heart Failure (reduced and preserved)	113 (22.2%)
	Coronary Artery Disease	109 (21.4%)
	Peripheral Arterial or Carotid Artery Disease	5 (1.0%)
	Chronic Kidney Disease	66 (13.0%)
	Arrhythmia	112 (22.0%)
	Thyroid Disease	101 (19.8%)
	Autoimmune Disease	81 (15.9%)

## Data Availability

Not applicable.

## Supplementary Materials

The following supporting information can be downloaded at: https://www.mdpi.com/article/10.3390/diseases10010005/s1, Table S1: Average selling price of medications used to treat PH.

## Abbreviations

CT	Computed tomography
CTEPH	Chronic thromboembolic pulmonary hypertension
DVT	Deep vein thrombus
ILD	Interstitial lung disease
mPAP	Mean pulmonary artery pressure
PAH	Pulmonary arterial hypertension
PE	Pulmonary embolus
PH	Pulmonary hypertension
PHCC	Pulmonary Hypertension Comprehensive Care Center
TTE	Transthoracic echocardiogram
RHC	Right heart catheterization
