# Supplementary material for: Comparing Diagnosis and Treatment of Pulmonary Hypertension Patients at a Pulmonary Hypertension Center versus Community Centers
Source: Diseases. 2022 Jan 7;10(1):5. doi: 10.3390/diseases10010005 (PMC8788556; doi:10.3390/diseases10010005)
Supplement: Supplementary file 1 [file diseases-10-00005-s001.zip › diseases-1493532-supplementary.pdf]

Article

Comparing Diagnosis and Treatment of Pulmonary Hypertension Patients at a Pulmonary Hypertension Center versus Community Centers

Hollie Saunders [1,*], Scott A. Helgeson [1], Ahmed Abdelrahim [1], Kathleen Rottman-Pietrzak [2], Victoria Reams [2], Tonya K. Zeiger [1], John E. Moss [1] and Charles D. Burger [1]

Table S1. Average selling price of medications used to treat PH.

Drug	Strength	Average Selling Price (ASP)	Average Daily Dose (ADD)	30 Day Supply	
				Quantity	Average Selling Price (ASP)
Epoprostenol (Flolan)	0.5 mg/vial	USD 22.43	2.52 mg	RT (8 h stable w sterile diluent): 540 vials	USD 12,112.20
				Ice pack (24 h stable w sterile diluent) or RT 95 °F (24 h stable w pH 12 sterile diluent: 180 vials	USD 4037.40
	1.5 mg/vial	USD 54.17		RT (8 h stable w sterile diluent): 180 vials	USD 9750.60
				Ice pack (24 h stable w sterile diluent) or RT 95 °F (24 h stable w pH 12 sterile diluent: 60 vials	USD 3250.20
Epoprostenol (Veletri)	0.5 mg/vial	USD 28.10	2.52 mg	180 vials	USD 5058.00
	1.5 mg/vial	USD 56.21		60 vials	USD 3372.60
Treprostinil (Remodulin)	200 mg/20 mL vial	Brand: USD 763.99 per mL<br><br>Generic: USD 716.24–725.79 per mL	5.04 mg	IV: 1 vial (200 mg/20 mL)<br>SQ: 1 vial (200 mg/20 mL)	Brand: USD 15,279.80<br><br>Generic: USD 14,324.8–14,515.80<br>Pure 30 d cost regardless of how supplied (cost per mL/30 day): USD 11,551.53 (Brand) and USD 10,829.55–10,973.95 (Generic)
Treprostinil inhaled (Tyvaso)	1.74 mg/2.9 mL ampule	USD 267.65 per mL	0.216 mcg (1 amp provides all daily doses)	30 ampules	8 packages (32 ampules): USD 24,837.92<br>Pure 30 d cost regardless of how supplied (cost per mL/30day): USD 23,285.55

Treprostinil oral (Orenitram)	0.125 mg/tab	USD 6.75 (per tab)	0.375 mg	60 tabs	USD 405.00
	0.25 mg/tab	USD 13.51 (per tab)	0.75 mg	90 tabs	USD 1215.90
	1 mg/tab	USD 54.02 (per tab)	3 mg		USD 4861.80
	2.5 mg/tab	USD 135.06 (per tab)	7.5 mg		USD 12,155.40
	5 mg/tab	USD 270.11 (per tab)	15 mg		USD 24,309.90
Iloprost inhaled (Ventavis)	10 mcg/mL ampule	USD 161.64 (per mL)	45 mcg (9 amps required)	270 ampules	USD 43,642.80
	20 mcg/mL ampule	USD 161.64 (per mL)	45 mcg (9 amps required)		USD 43,642.80
Bosentan (Tracleer)	62.5 mg/tab	Brand: USD 232.63 (per tab)<br><br>Generic: USD 23.26–221.00 (per tab)	125 mg	60 tabs	Brand: USD 13,957.80<br>Generic: USD 1395.60–13,260.00
	125 mg/tab	Brand: USD 232.63 (per tab)<br><br>Generic: USD 23.26–221.00 (per tab)	250 mg		Brand: USD 13,957.80<br><br>Generic: USD 1395.60–13,260.00
Macitentan (Opsumit)	10 mg/tab	USD 403.50 (per tab)	10 mg	30 tabs	USD 12,105.00
Ambrisentan (Letairis)	5 mg/tab	Brand: USD 406.96 (per tab)<br><br>Generic: USD 242.67–368.91 (per tab)	5 mg	30 tabs	Brand: USD 12,208.80<br><br>Generic: USD 7280.10–11,067.30
	10 mg/tab	Brand: USD 406.96 (per tab)<br><br>Generic: USD 242.67–368.91 (per tab)	10 mg	30 tabs	Brand: USD 12,208.80<br><br>Generic: USD 7280.10–11,067.30
Selexipag (Uptravi)	200 mcg/tab	USD 236.06 (per tab)	400 mcg	60 tabs	USD 14,163.60
	400 mcg/tab	USD 367.12 (per tab)	800 mcg		USD 22,027.20
	600 mcg/tab	USD 367.12 (per tab)	1200 mcg		USD 22,027.20
	800 mcg/tab	USD 367.12 (per tab)	1600 mcg		USD 22,027.20
	1000 mcg/tab	USD 367.12 (per tab)	2000 mcg		USD 22,027.20

	1200 mcg/tab	USD 367.12 (per tab)	2400 mcg		USD 22,027.20
	1400 mcg/tab	USD 367.12 (per tab)	2800 mcg		USD 22,027.20
	1600 mcg/tab	USD 367.12 (per tab)	3200 mcg		USD 22,027.20
Sildenafil (Revatio)	20 mg/tab	USD 60.04 (per tab)	60 mg	60 tabs	USD 3602.40
Tadalafil (Adcirca)	20 mg/tab	Brand: USD 83.40 (per tab)<br><br>Generic: USD 72.14–76.15 (per tab)	20 mg	60 tabs	Brand: USD 5004.00<br><br>Generic: USD 4328.40–4569
Riociguat (Adempas)	0.5 mg/tab	USD 146.11 (per tab)	1.5 mg	90 tabs	USD 13,149.90
	1 mg/tab	USD 146.11 (per tab)	3 mg		USD 13,149.90
	1.5 mg/tab	USD 146.11 (per tab)	4.5 mg		USD 13,149.90
	2 mg/tab	USD 146.11 (per tab)	6 mg		USD 13,149.90
	2.5 mg/tab	USD 146.11 (per tab)	7.5 mg		USD 13,149.90

Lexicomp. (n.d.) Drug information. UpToDate. Retrieved 24 January 2020 from *http://www.uptodate.com*.
